# Immediate versus staged complete revascularization in patients with acute coronary syndrome and multivessel disease: a meta-analysis of randomized controlled trials

**DOI:** 10.3389/fcvm.2025.1626748

**Published:** 2025-09-29

**Authors:** Lin He, Qing-Juan Yang, Bin Sun, Cheng Guo, Ji-Ling Hu, Hong-Pie Li, Jing-Hong Zhao, Peng-Yu Zhong

**Affiliations:** ^1^ Department of Cardiology, Beijing Anzhen Nanchong Hospital of Capital Medical University and Nanchong Central Hospital (The Second Clinical College of North Sichuan Medical College), Nanchong, Sichuan, China; ^2^ Xihua University, Chengdu, Sichuan, China

**Keywords:** percutaneous coronary intervention, acute coronary syndrome, immediate complete revascularization, staged complete revascularization, multivessel disease (MVD)

## Abstract

**Background:**

A series of trials have confirmed that complete revascularization is more beneficial for patients with acute coronary syndrome (ACS) and multivessel disease than culprit-only revascularization. However, the optimal timing of complete revascularization remains controversial. It is unclear whether immediate complete revascularization is safer and more effective than staged complete revascularization.

**Method:**

This meta-analysis of randomized controlled trials aimed to compare the efficacy and safety of immediate vs. staged revascularization in patients with ACS. The primary outcome was major adverse cardiovascular events (MACE), which were defined as a composite endpoint. Risk ratios (RRs) were calculated using the Mantel–Haenszel (M-H) fixed-effect model. Trial sequential analysis was additionally performed to validate the results. This study is registered with PROSPERO (CRD42023461852).

**Results:**

In total, 11 randomized studies involving 5,666 patients met the inclusion criteria. At a mean follow-up of 16 months, immediate complete revascularization significantly decreased the incidence of MACE compared with staged complete revascularization [RR: 0.76, 95% confidence interval (CI): 0.66–0.89, *P* = 0.0004]. Significant decreases were also observed in repeat myocardial infarction (RR: 0.59, 95% CI: 0.43–0.82, *P* = 0.002), repeat revascularization (RR: 0.62, 95% CI: 0.48–0.79, *P* = 0.0001), and the composite outcome of myocardial infarction or death (RR: 0.67, 95% CI: 0.48–0.92, *P* = 0.01). However, no significant differences were found in all-cause mortality (RR: 0.92, 95% CI: 0.64–1.33, *P* = 0.66) or cardiovascular mortality (RR: 0.96, 95% CI: 0.58–1.61, *P* = 0.89).

**Conclusion:**

In patients with ACS and multivessel disease, immediate complete revascularization significantly decreased the risk of MACE, repeat myocardial infarction, and repeat revascularization, without increasing the risk of all-cause death.

## Introduction

Acute coronary syndrome (ACS) is a common cardiovascular disease caused by insufficient blood supply to the coronary arteries. This insufficient blood supply can lead to myocardial infarction (MI) or unstable angina. Multivessel disease (MVD), defined as the presence of significant stenoses or occlusions in multiple coronary arteries, is frequently encountered in ACS, with approximately 50% of patients with ST-segment elevation myocardial infarction (STEMI) found to have MVD on initial coronary angiography (1). Compared to those with single-vessel disease, these patients have higher short- and long-term mortality rates (2–4). Percutaneous coronary intervention (PCI) has emerged as a cornerstone therapy for STEMI, significantly improving prognoses and providing net clinical benefits (2). Numerous studies have now demonstrated that complete revascularization (CR) is superior to infarct-related vessel revascularization alone and can significantly reduce the risk of recurrent myocardial infarction in these patients without cardiogenic shock (5–9). Contemporary developments in coronary intervention have further improved the prognosis of these patients, particularly through the application of extracorporeal membrane oxygenation.

Patients who have undergone immediate complete revascularization (ICR) have achieved good short-term outcomes with higher rates of coronary revascularization, faster postoperative recovery, and a lower complication rate. However, this treatment strategy is technically demanding, requires an experienced medical team, and may increase the risk of operative time and postoperative recovery. Comparatively, patients who undergo staged complete revascularization (SCR) may need to undergo multiple procedures and have a longer treatment course. However, this strategy is less risky and less physically taxing on the patient. Moreover, this treatment approach allows for a gradual recovery through the different surgical phases, reduces the load on the heart, and is safer for elderly or frail patients. The current guidelines recommend CR for patients with ACS and MVD who are free of cardiogenic shock (10–12). However, the optimal timing for treating non-culprit lesions in this population remains undefined. The European Society of Cardiology (ESC) guidelines recommend that patients with ACS combined with multibranch vasculopathy should undergo CR within 45 days (12). However, it is controversial whether patients who undergo ICR gain any additional benefit.

Based on previous research, this study aimed to include relevant randomized controlled trials (RCTs) to explore the efficacy and safety of ICR compared with SCR. In addition, a trial sequential analysis (TSA) was used to assess the outcomes.

## Method

### Data sources and inclusion and exclusion criteria

We conducted this meta-analysis in accordance with the Preferred Reporting Items for Systematic Reviews and Meta-Analysis (PRISMA) guidelines (13). A comprehensive search without language restrictions was performed in PubMed, Embase, and the Cochrane Library from database inception to 1 March 2025. The PubMed search strategy included the following keywords: “ST-elevation myocardial infarction”, “Multivessel disease”, “complete revascularization”, “staged revascularization”, “simultaneous revascularization”, “culprit only revascularization”, “infarct-related artery revascularization”, and “randomized controlled trial”. Detailed search terms are provided in Supplementary Table S1.

In addition to electronic database searches, the reference lists of the included studies were manually screened. Conference proceedings from major cardiology societies (e.g., American Heart Association, American College of Cardiology, Transcatheter Cardiovascular Therapeutics, European Society of Cardiology, and Congress of the European Association of Percutaneous Cardiovascular Interventions) were also reviewed for relevant abstracts. The selection process comprised the following two stages: (1) the initial exclusion of irrelevant studies based on title/abstract review and (2) a detailed assessment of potentially eligible studies.

Two reviewers (LH and BS) independently evaluated each study’s eligibility, with discrepancies resolved by a third investigator (P-YZ). Studies were included if they met all the following criteria: involving patients with ACS, including STEMI, unstable angina, or non-STEMI; comparing ICR and SCR; reporting predefined clinical outcomes (e.g., cardiovascular events); and being an RCT. Studies were excluded if they lacked a valid control group or relevant cardiovascular/cerebrovascular outcome data or were non-original publications (e.g., reviews, editorials, commentaries). Non-English studies were translated using professional translation services or software when necessary.

### Data extraction and outcome assessments

Two authors (LH and BS) independently extracted data from eligible studies using piloted data extraction sheets. The extracted data covered aspects including the first author, publication year, study setting, follow-up duration, study design, sample size, and the personal and clinical characteristics of the participants.

The primary outcome was defined as major adverse cardiovascular events (MACE). The definition of MACE was the same as that used in the original study. For RCTs with multiple definitions, we selected the primary MACE outcome that was consistent with those in the other RCTs. For the efficacy evaluation, the outcomes included were repeat myocardial infarction and repeat revascularization. Regarding the safety assessment, the outcomes included all-cause mortality, cardiovascular mortality, and the composite outcome of death or myocardial infarction.

### Risk of bias and certainty of evidence assessment

Two researchers (LH and BS) independently evaluated the risk of bias of the included studies using the Risk of Bias 2 (RoB 2) tool (14, 15). Two reviewers (LH and BS) assessed the risk level of each study to be low, moderate, serious, critical, or no information. Detailed descriptions and decision criteria for each ROB 2 domains are provided in Supplementary Appendix S4. Discrepancies were resolved by a senior investigator (J-HZ).

Two investigators (Q-JY and J-LH) independently appraised the evidence certainty for each outcome, with disagreements adjudicated by a third reviewer (P-YZ). The Grading of Recommendations Assessment, Development, and Evaluation (GRADE) framework was employed to evaluate the evidence certainty, categorizing it into the following levels: very low, low, moderate, and high (16).

### Statistical analysis

We conducted the statistical analyses using Review Manager (version 5.4). The effect size was measured as relative risk with 95% confidence intervals (95% CIs).

The judgment of heterogeneity was based on Cochran's *Q*-test. When *P* ≥ 0.1, no significant heterogeneity was considered to exist and the Mantel–Haenszel (M-H) fixed-effects model was used. When *P* < 0.1, significant heterogeneity was deemed to exist and the degree of heterogeneity was then evaluated using I^2^. Thus, I^2^ < 25%, 25%–50%, and >50% were respectively categorized as low, moderate, and high heterogeneity levels (17), respectively. Subgroup analyses were performed based on myocardial infarction subtype, timing of SCR, and definition of MACE. Publication bias was initially assessed via visual inspections of the funnel plots and Egger's test. To minimize the risk of type I errors caused by repeated significance testing or an insufficient sample size, we conducted a TSA to assess the robustness of the pooled effect. The TSA was performed using TSA software (version 0.9.5.10), employing a two-sided testing model with a type I error (α) of 0.05 and a statistical power (1 − β) of 80%. The relative risk reduction (RRR) was estimated based on data from recently published large-scale RCTs (BIOVASC, MULTISTARS AMI, and Wood et al.), and the required information size (RIS) was calculated accordingly. If the cumulative Z-curve crossed the TSA monitoring boundary or the RIS was reached with the Z-curve exceeding the conventional significance threshold, the evidence was considered sufficient and the result statistically robust.

## Results

### Search results and baseline characteristics

**Figure 1:**
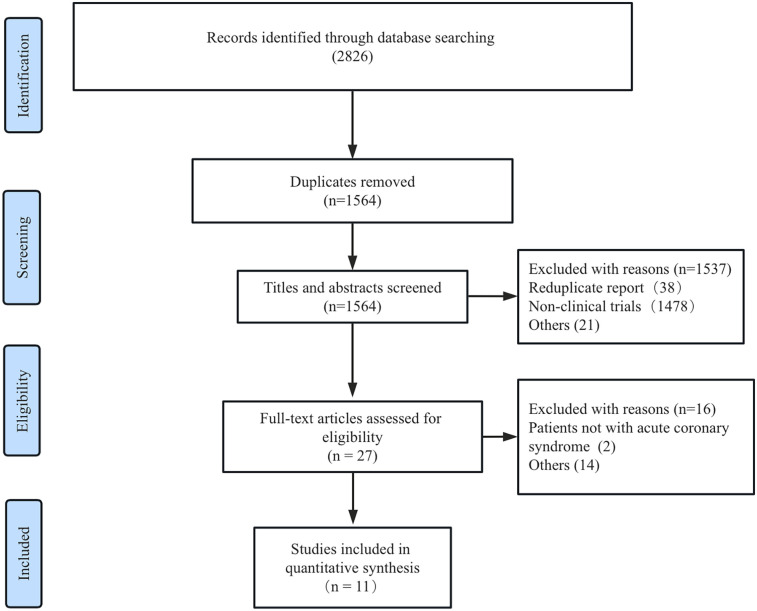
Flow diagram of the literature search.

The literature screening and study selection process is depicted in Figure 1. A total of 2,826 studies were initially retrieved from the PubMed, Embase, and Cochrane Library databases. After reviewing 27 full-text articles, 11 RCTs ultimately met the predefined inclusion criteria (18–27).

Table 1 outlines the characteristics of the included trials. The majority of studies were single-center RCTs involving patients with STEMI without cardiogenic shock. A total of eight RCTs enrolled patients with STEMI, two RCTs only included patients with non-STEMI, and only one RCT (BIOVASC) included patients with unstable angina. The timing of SCR ranged from 2 to 45 days, and the follow-up durations varied from 6 months to 4 years. The baseline patient characteristics are presented in Table 2, with no significant differences in clinical presentations observed between the ICR and SCR groups.

**Table 1:** Baseline characteristics of the included trials.

Trials	Type of ACS	Single/multi-center	ICR/SCR	Timing of SCR (days)	Exclusion criteria	MACE	Follow-up
PRIMA (Ochala et al., 2004)	STEMI	Single	48/44	<7	Cardiogenic shock	AD, MI, TVR	6 months
Politi et al., 2010	STEMI	Single	65/65	20–45	Cardiogenic shock; left main stenosis; previous CABG; severe valvular heart disease	AD, MI, IDR, rehospitalization	30 months
Maamoun et al., 2011	STEMI	Single	42/36	<7	Cardiogenic shock; pulmonary edema; left main stenosis	AD, MI, TVR, CVA, rehospitalization	12 months
Tarasov et al., 2013	STEMI	Single	46/43	8.5 ± 4.2	Acute heart failure Killip III–IV; ≥50% left main stenosis	AD, MI, IDR	6 months
SMILE (Sardella et al., 2016)	Non-STEMI	Single	264/263	4.8 ± 1.2	Cardiogenic shock; chronic total occlusion; previous CABG; severe valvular disease	AD, MI, TVR, stroke, rehospitalization	12 months
BIOVASC (Diletti et al., 2023)	ACS	Multi-center	764/761	30–42	Previous CABG, cardiogenic shock, and CTO	AD, MI, IDR, CVA	12 months
MULTISTARS AMI	STEMI	Multi-center	418/422	19–45	Previous CABG, cardiogenic shock, and CTO	AD, MI, IDR, stroke, rehospitalization	12 months
Park et al., 2023	STEMI	Multi-center	103/106	4.4 (1–11)	History of bleeding diathesis or known coagulopathy; LVEF <25% or presence of cardiogenic shock	AD, MI, IDR	12 months
Elkady et al., 2021 (35)	Non-STEMI	Single	30/30	20–42	Previous CABG, cardiogenic shock, and CTO	AD, MI, IDR, rehospitalization	6 months
Brendea et al., 2021 (36)	STEMI	Single	50/50	2–3	Previous CABG and cardiogenic shock	AD, MI, IDR, stroke	24 months
Wood et al., 2019	STEMI	Multi-center	1,353/663	<45	Previous CABG, cardiogenic shock	CD, MI, IDR	4 years

ACS, acute coronary syndrome; PCI, percutaneous coronary intervention; STEMI, ST-elevation myocardial infarction; CABG, coronary artery bypass grafting; CD, cardiovascular death; CTO, chronic total occlusion; AD, all-cause death; MI, myocardial infarction; IDR, unplanned ischemia-driven revascularization; CVA, cerebrovascular accident; LVEF, left ventricular ejection fraction; TVR, target vessel revascularization.

**Table 2:** Baseline characteristics of the included patients.

Trials	Age	Male (%)	Diabetes (%)	Hypertension (%)	Smoking (%)	Hyperlipidemia (%)	Anterior myocardial infarction
PRIMA (Ochala et al., 2004)	65/67	73/75	31/34	52/48	36/43	81/91	46/45
Politi et al., 2010	65/64	77/80	14/19	49/65	NR	NR	48/49
Maamoun et al., 2011	55/52	95/89	42/57	38/33	52/57	57/44	62/69
Tarasov et al., 2013	59/59	70/58	26/21	96/86	NR	NR	46/30
SMILE (Sardella et al., 2016)	72/73	78/79	34/35	73/66	45/41	58/54	71/72
BIOVASC (Diletti et al., 2023)	66/65	78/77	21/21	58/52	52/51	51/53	66/63
MULTISTARS AMI	66/64	77/81	16/15	55/50	53/49	27/27	40/41
Park et al., 2023	63/62	80/83	41/35	51/45	52/53	37/39	55/60
Elkady et al., 2021 (35)	NR	NR	NR	NR	NR	NR	NR
Brendea et al., 2021 (36)	NR	37/36	12/11	20/24	25/21	NR	22/18
Wood et al., 2019	62/61	80/82	20/18	50/46	3/4	38/38	NR

The data shown are for the ICR/SCR groups.

NR, Not reported.

### Primary outcome

**Figure 2:**
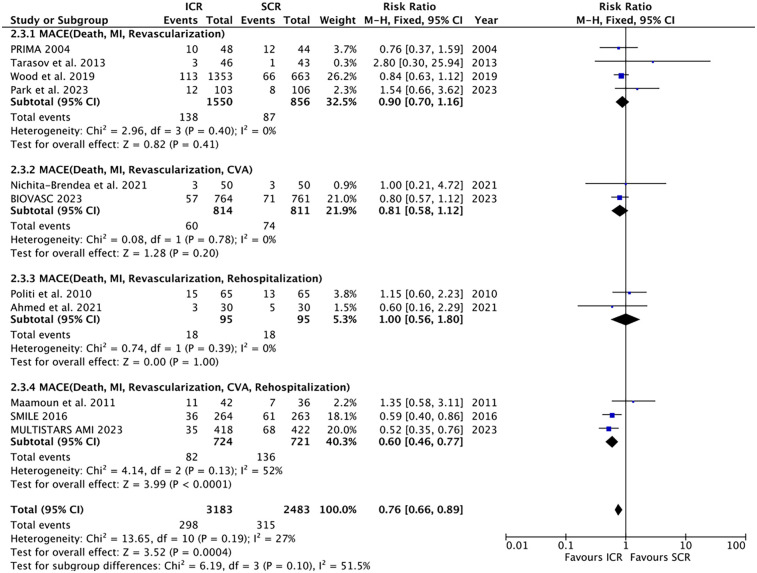
Pooled analyses of ICR compared to SCR for the primary outcomes.

All the included trials reported the incidence of MACE (Figure 2). The results showed that ICR significantly decreased the incidence of MACE compared to SCR (RR 0.76, 0.66–0.89, *P* = 0.0004, I^2^ = 27%, *P*_heterogeneity_ = 0.19). Further subgroup analyses were conducted based on differences in MACE definitions, and the results showed no significant differences in the following subgroups: MACE defined as death, MI, or revascularization (RR 0.90, 95% CI 0.70–1.16, *P* = 0.41; I^2^ = 0%, *P*_heterogeneity_ = 0.40); MACE defined as death, MI, revascularization, or cerebrovascular accident (CVA) (RR 0.81, 95% CI 0.58–1.12, *P* = 0.20; I^2^ = 0%, *P*_heterogeneity_ = 0.78); MACE defined as death, MI, revascularization, or rehospitalization (RR 1.00, 95% CI 0.56–1.80, *P* = 1.00; I^2^ = 0%, *P*_heterogeneity_ = 0.39). In contrast, ICR significantly decreased the risk of MACE (death, MI, revascularization, CVA, or rehospitalization) compared to SCR (RR 0.60, 95% CI 0.46–0.77, *P* < 0.0001, I^2^ = 52%, *P*_heterogeneity_ = 0.13).

In the subgroup analysis according to the difference in SCR timing (Supplementary Figure S2), ICR significantly decreased the risk of MACE (RR 0.70, 0.56–0.88, *P* = 0.003) compared to SCR at 14–45 days. However, no difference was found in ICR compared with SCR at <14 days.

The subgroup analysis by myocardial infarction type (Supplementary Figure S3) revealed that in the STEMI subgroup, ICR was associated with a lower incidence of MACE compared to SCR (RR 0.81, 95% CI 0.67–0.97, *P* = 0.02). This was consistent with the results observed in the non-STEMI subgroup (RR 0.67, 95% CI 0.51–0.88, *P* = 0.004).

### Repeat myocardial infarction and repeat revascularization

**Figure 3:**
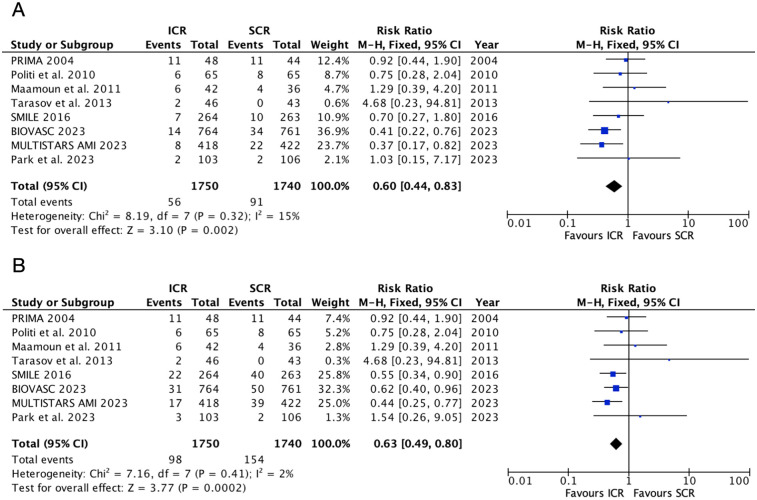
Pooled analyses of ICR compared to SCR for the efficacy outcomes. **(A)** Repeat myocardial infarction and **(B)** repeat revascularization.

Seven trials reported the repeat myocardial infarction and repeat revascularization outcomes (Figure 3). The risk of repeat myocardial infarction decreased by 40% in the ICR group compared with SCR, with low heterogeneity (RR 0.60, 0.44–0.83, *P* = 0.002, I^2^ = 15%, *P*_heterogeneity_ = 0.32). Similarly, ICR also decreased the risk of repeat revascularization by 37% compared with the SCR group (RR 0.63, 0.49–0.80, *P* = 0.002, I^2^ = 2%, *P*_heterogeneity_ = 0.41).

Further subgroup analysis based on the timing of SCR (Supplementary Figure S2) showed that, when compared with SCR at <14 days, ICR did not decrease the risk of repeat myocardial infarction (RR 0.97, 95% CI 0.59–1.59, *P* = 0.91) and repeat revascularization (RR 0.75, 95% CI 0.52–1.07, *P* = 0.12). However, when compared with SCR at 14–45 days, ICR significantly decreased the risk of repeat revascularization (RR 0.44, 95% CI 0.28–0.68, *P* = 0.0002) and repeat revascularization (RR 0.56, 95% CI 0.40–0.77, *P* = 0.0004). Significant heterogeneity was observed between these subgroups (*P* = 0.02) for the repeat myocardial infarction outcome, but no heterogeneity was observed for the repeat revascularization outcome (*P* = 0.24).

A further subgroup analysis based on myocardial infarction type (Supplementary Figure S3) showed that only one study was included in the non-STEMI subgroup. In the STEMI subgroup, when compared with SCR, ICR did not decrease the risk of repeat myocardial infarction (RR 0.63, 95% CI 0.49–0.80, *P* = 0.002) but significantly decreased the risk of repeat revascularization (RR 0.63, 95% CI 0.49–0.80, *P* = 0.002).

### All-cause mortality, death or myocardial infarction, and cardiovascular mortality

**Figure 4:**
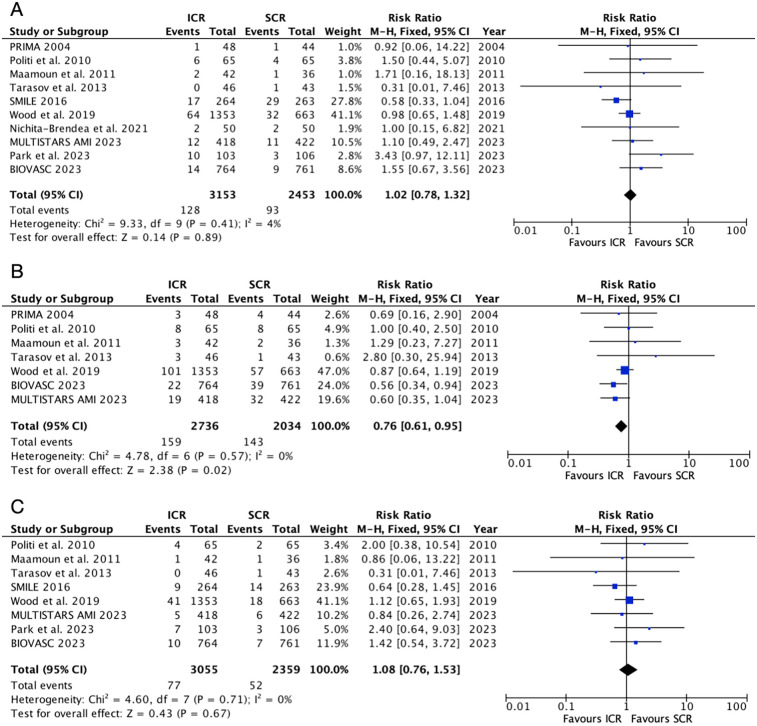
Pooled analyses of ICR compared to SCR for the safety outcomes. (**A**) All-cause mortality, (**B**) death or myocardial infarction, and (**C**) cardiovascular mortality.

The safety outcomes were shown in Figure 4. Six randomized controlled trials included in the all-cause mortality outcome, which showed that ICR did not increase the risk of all-cause mortality compared to SCR (RR: 1.02, 95% CI: 0.78–1.32, *P* = 0.89, *P*_heterogeneity_ = 0.41, I^2^ = 4%). Similarly, ICR was not associated with a higher risk of cardiovascular mortality compared to SCR (RR: 1.08, 95% CI: 0.76–1.53, *P* = 0.67, *P*_heterogeneity_ = 0.71, I^2^ = 0%). However, ICR decreased the risk of death or myocardial infarction by 24% compared to SCR, without any heterogeneity (RR: 0.76, 95% CI: 0.61–0.95, *P* = 0.02, *P*_heterogeneity_ = 0.57, I^2^ = 0%). A further subgroup analysis based on the timing of SCR and myocardial infarction type (Supplementary Figures S2, S3) showed no difference between the two subgroups for all-cause mortality and cardiovascular mortality. ICR was associated with a lower risk of death or myocardial infarction (RR: 0.62, 95% CI: 0.44–0.88, *P* = 0.007) compared to SCR at 14–45 days. However, no significant difference was observed between ICR and SCR at <14 days (RR: 1.16, 95% CI: 0.45–3.02, *P* = 0.76).

### Publication bias and assessment of quality

The publication bias analyses resulted in asymmetrically distributed funnel plots for all the outcomes (Supplementary Figure S4). The Egger's tests, the results of which are presented in Supplementary Table S2, indicated that for each of the outcomes, no significant publication bias was found, as evidenced by all the *P*-values being greater than 0.05. The quality assessment of each trial and the GRADE evidence evaluations are detailed in Supplementary Table S3. All the included trials exhibited a low risk of bias across the selection, detection, performance, and reporting domains. The GRADE assessments confirmed moderate to high certainty for all the evaluated outcomes.

### Trial sequential analysis

**Figure 5:**
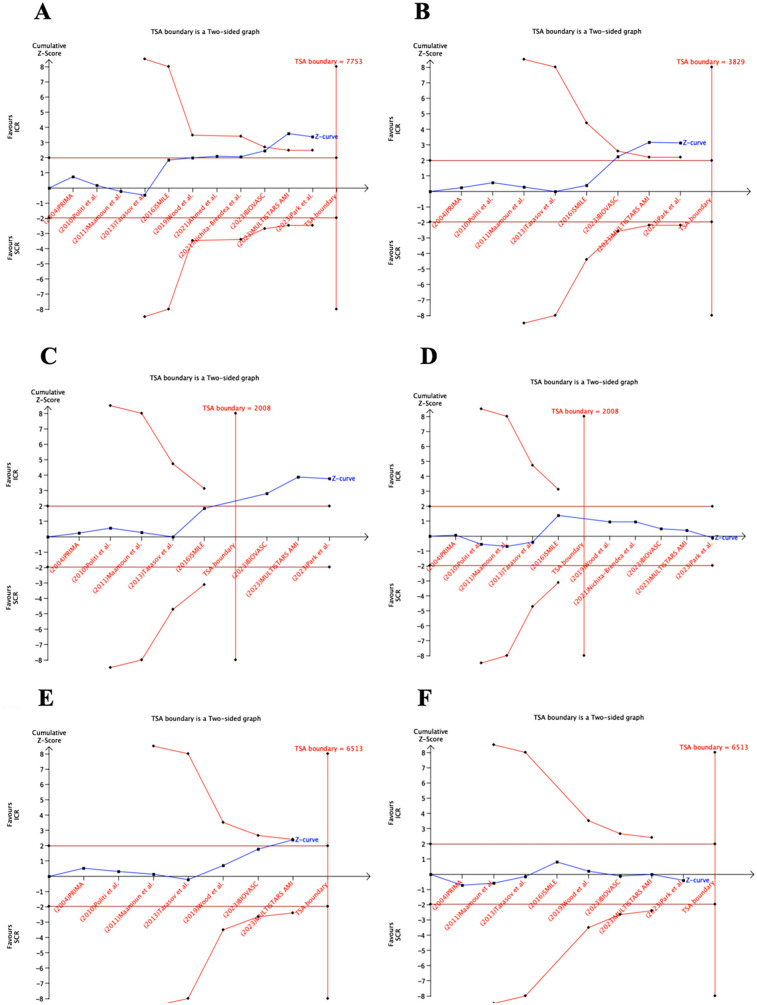
TSA of the studies that compared ICR and SCR. RIS, required information size. ICR, immediate complete revascularization; SCR, staged complete revascularization; TSA, trial sequential analysis; I have comfimed RIS, required information size.

The TSA results are presented in Figure 5. For MACE, repeat myocardial infarction, and repeat revascularization, the cumulative z-curves crossed the conventional statistical significance boundaries and reached the RIS. For all-cause death, the z-curve did not cross the conventional statistical significance boundaries but still achieved the RIS. For the composite outcome of death or myocardial infarction, the z-curve crossed both the conventional and TSA boundaries. However, for cardiovascular death, the z-curve did not cross either the conventional or TSA boundaries, and an RIS of 6,513 would be required to robustly address this outcome.

## Discussion

ICR was associated with a lower risk of MACE, repeat myocardial infarction, repeat revascularization, and death or myocardial infarction compared to SCR. Moreover, ICR did not increase the risk of all-cause mortality and cardiovascular mortality.

MVD is highly prevalent in patients with ACS, with these individuals having a higher risk of mortality and poorer clinical outcomes compared to those with single-vessel disease (28). Multiple RCTs have demonstrated that complete revascularization provides greater net clinical benefits for patients with ACS and MVD than culprit vessel revascularization alone (5–9). The efficacy of ICR has been validated in several RCTs. The BIOVASC trial, a multi-center randomized study involving 1,525 patients with ACS across 29 hospitals, compared ICR and SCR (22). The results showed that ICR significantly reduced the incidence of myocardial infarction [hazard ratio (HR) 0.41, 95% CI 0.22–0.76, *P* = 0.0045] and unplanned ischemia-driven revascularizations (HR 0.61, 95% CI 0.39–0.95, *P* = 0.030). However, no significant effect was observed for all-cause mortality (HR 1.56, 95% CI 0.68–3.61, *P* = 0.30). Similarly, the MULTISTARS AMI trial found that immediate multivessel PCI was non-inferior to staged multivessel PCI in reducing the risk of composite outcomes, including all-cause death, fatal/non-fatal myocardial infarction, stroke, and heart failure-related hospitalizations, within 1 year (23).

The net clinical benefit of ICR in hemodynamically stable patients with STEMI and MVD remains unclear. The 2023 ESC guidelines for ACS management recommend culprit vessel revascularization (Class Ia) during primary PCI, followed by SCR (Class IIa) for patients with MVD and cardiogenic shock. In addition, patients with STEMI without cardiogenic shock are advised to undergo complete revascularization either during the initial procedure or within 45 days (12).

ICR can restore the blood supply at an early stage. This can confer multiple benefits, such as reducing the incidence of thrombotic events in the acute phase, improving early cardiac function, and cutting medical costs (29). However, ICR may increase the use of contrast agents and prolong the operating time. Thus, these disadvantages must be weighed against the increased risk of periprocedural myocardial infarction, particularly during complex multivessel PCI (30). However, acute myocardial infarction can cause microangiopathy, which diminishes the vasodilatory response. This situation can affect the assessment of non-culprit vessels, leading to further overestimation of the degree of stenosis and resulting in more stent implantations. Therefore, SCR can reduce the risk associated with primary PCI and allow for a more accurate assessment of non-culprit vessels.

In patients with STEMI, the culprit lesion can be distinctly identified based on an ECG. However, in non-ST-segment elevation acute coronary syndromes, ECG-based identification may be misleading. Misidentification of the culprit lesion could lead to treatment of a non-culprit lesion (31–33). In the context of acute coronary syndromes, non-culprit lesions may also exhibit unstable characteristics. These characteristics make them prone to plaque rupture and the development of acute coronary syndromes within the time interval between immediate and staged surgery (27). Both of these scenarios may support an ICR strategy. Furthermore, they help explain the 41% decrease in the risk of myocardial infarction and the 38% decrease in the risk of unplanned ischemia-driven revascularization observed in our study.

Zhou et al. conducted a meta-analysis that compared ICR and SCR in patients with MDV, which was similar to our study (34). The results showed that ICR decreased the risk of MACE, myocardial infarction, and repeat revascularization by 27%, 47%, and 36%, respectively. All-cause mortality, cardiovascular death incidence, and stroke incidence did not significantly differ between the two groups. The findings of Zhou et al. are consistent with ours. Our study further conducted subgroup analyses based on the timing of SCR and the type of myocardial infarction. Compared with SCR performed within 14 days, ICR did not reduce the risk of MACE, myocardial infarction, or repeat revascularization. This suggests that the effectiveness of ICR may be more evident when compared with SCR performed between 14 and 45 days. Therefore, in clinical practice, both ICR and SCR performed within 14 days may be preferable options. Furthermore, a total of 11 RCTs were included in this article, among which eight RCTs enrolled patients with STEMI, two RCTs included only patients with non-STEMI, and only one RCT (BIOVASC) included patients with unstable angina. Therefore, the included studies focused more on patients with myocardial infarction, and the research conclusions were consistent for the patients with STEMI or non-STEMI. For unstable angina, however, more clinical trial data are needed, and the current conclusions cannot be directly applied to this patient population.

Based on the TSA, ICR can adequately reduce the risk of MACE, repeat myocardial infarction, repeat revascularization, all-cause death, and death or myocardial infarction. Further RCTs are not required to demonstrate this. Furthermore, the all-cause death outcome has met the RIS, and I have comfimed this finding likely reflects a true negative result. 

## Conclusions

In patients with ACS and MVD, ICR significantly decreases the risk of MACE, repeat myocardial infarction, and repeat revascularization, with no associated increase in all-cause mortality. This favorable effect is especially pronounced when compared with SCR administered between 14 and 45 days.

## Limitations

This meta-analysis of randomized controlled trials had several limitations. First, it was based on study-level data rather than individual patient data, which may have limited the depth of the subgroup analyses and the adjustments for potential confounders. Second, the majority of included trials were open-label, which could have introduced performance bias, particularly in the outcomes that were assessed subjectively. Third, the timing of SCR ranged from a few days to 45 days, which increased the heterogeneity of the studies, making it difficult to determine a uniform optimal treatment regimen. Finally, there were large differences in the prevalence of risk factors for coronary heart disease, namely, diabetes, hypertension, and hyperlipidemia, between the two groups, which may have contributed to the differences in outcomes.

## Data Availability

The original contributions presented in the study are included in the article/Supplementary Material, further inquiries can be directed to the corresponding authors.
