# Supplementary material for: Immediate versus staged complete revascularization in patients with acute coronary syndrome and multivessel disease: a meta-analysis of randomized controlled trials
Source: Front Cardiovasc Med. 2025 Sep 29;12:1626748. doi: 10.3389/fcvm.2025.1626748 (PMC12515846; doi:10.3389/fcvm.2025.1626748)
Supplement: Supplementary file 1 [file Datasheet1.pdf]

Table S1. Search strategy and results in PubMed database.

#	Searches	Results
**#1**	"acute coronary syndrome"[Mesh] OR "percutaneous coronary intervention"[Mesh] OR "coronary intervention*, percutaneous":[tiab] OR "intervention*, percutaneous coronary":[tiab] OR "pci":[tiab] OR "percutaneous coronary revascularization":[tiab] OR "coronary revascularization*, percu -taneous":[tiab] OR "percutaneous coronary revascularizations": [tiab] OR "revascularization*, percutaneous coronary":[tiab] OR "drug eluting stent*":[tiab] OR "des":[tiab] OR "bare metal stent":[tiab] OR "BMS":[tiab]	**111553**
**#2**	"aspirin":[tiab] OR "clopidogrel":[tiab] OR "prasugrel":[tiab] OR "ticagrelor":[tiab] OR "p2y12 inhibitor":[tiab]	**59031**
**#3**	"Randomized Controlled Trial"[Publication Type] OR "controll -ed clinical trial":[tiab] OR "clinical trials, randomized":[tiab] OR "trials, randomized clinical":[tiab] OR "randomized clinical studies":[tiab]	**534219**
**#4**	"Systematic Review"[Publication Type] OR "Meta-Analysis" [Publication Type] OR "Review"[Publication Type] OR "meta-analysis":[ti] OR "systematic review":[ti] OR "literature review" :[ti] OR "expert consensus":[ti] OR "case report":[ti] OR "dual antiplatelet therapy":[ti] OR "atrial fibrillation":[ti]	**3220842**
**#5**	**#1 AND #2 AND #3 NOT #4**	**1003**

Table S2. Subgroup analysis of MACE

Subgroup	Grouping Status	Number of studies	Meta-analysis			Heterogeneity		P-value of difference between subgroups
			Effect size	95%CI	P-value	I^2	P-value	
Signal	single	7	0.77	0.59-1.00	P=0.05	10%	P=0.35	0.86
	Multi	4	0.79	0.67-0.93	P=0.006	31%	P=0.15	
Type	STEMI	8	0.84	0.70-0.99	P=0.04	39%	P=0.12	0.24
	ACS	3	0.70	0.55-0.89	P=0.004	0%	P=0.47	
Follow-up time	≥12	8	0.78	0.68-0.91	P=0.0009	47%	P=0.07	0.85
	<12	3	0.83	0.44-1.53	P=0.56	0%	P=0.49	

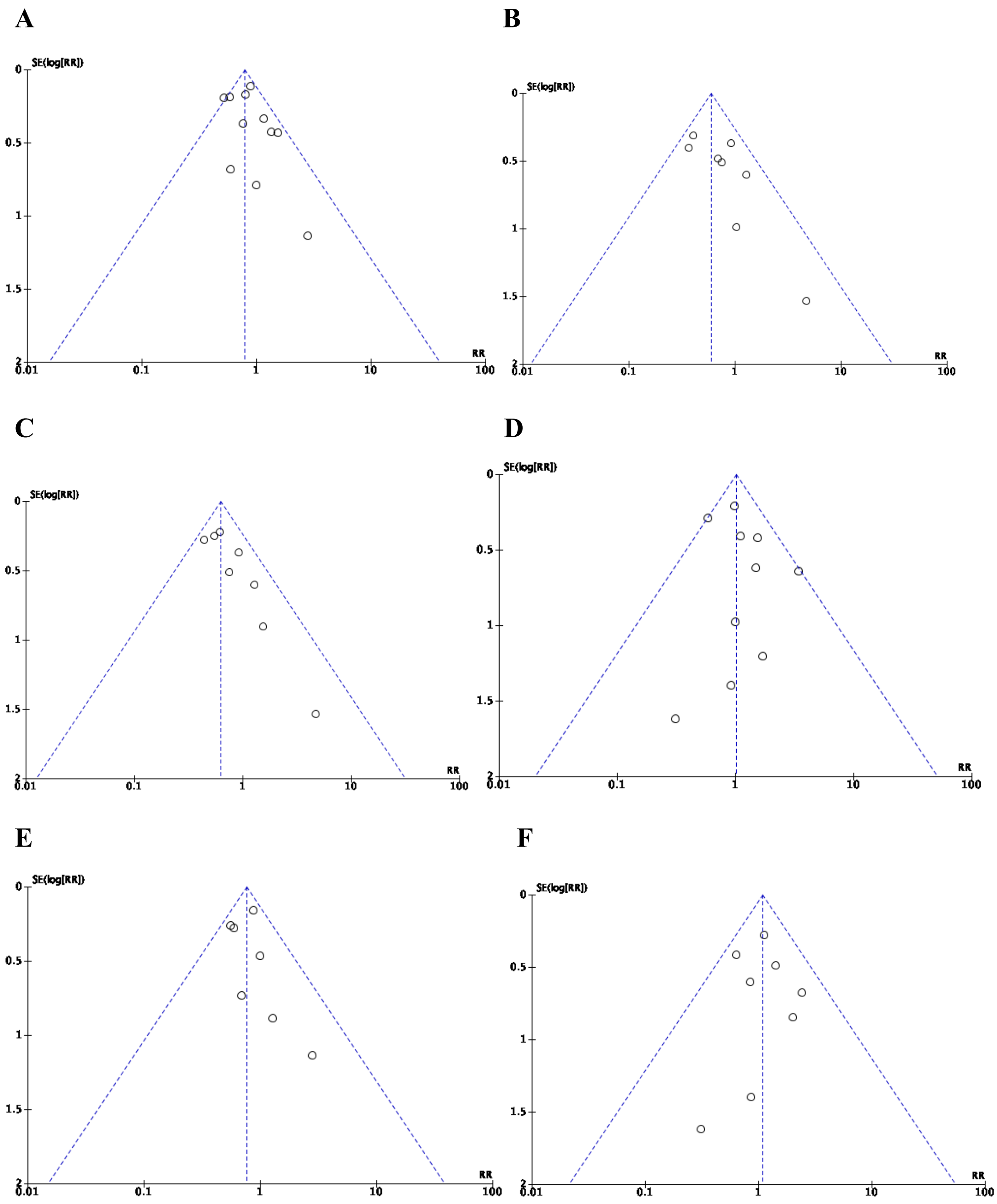

Figure S1. funnel plots.

A. MACE, B.Repeat myocardial infarction; C.repeat revascularization D.all-cause mortality, E.death or myocardial infarction, F.cardiovascular mortality.

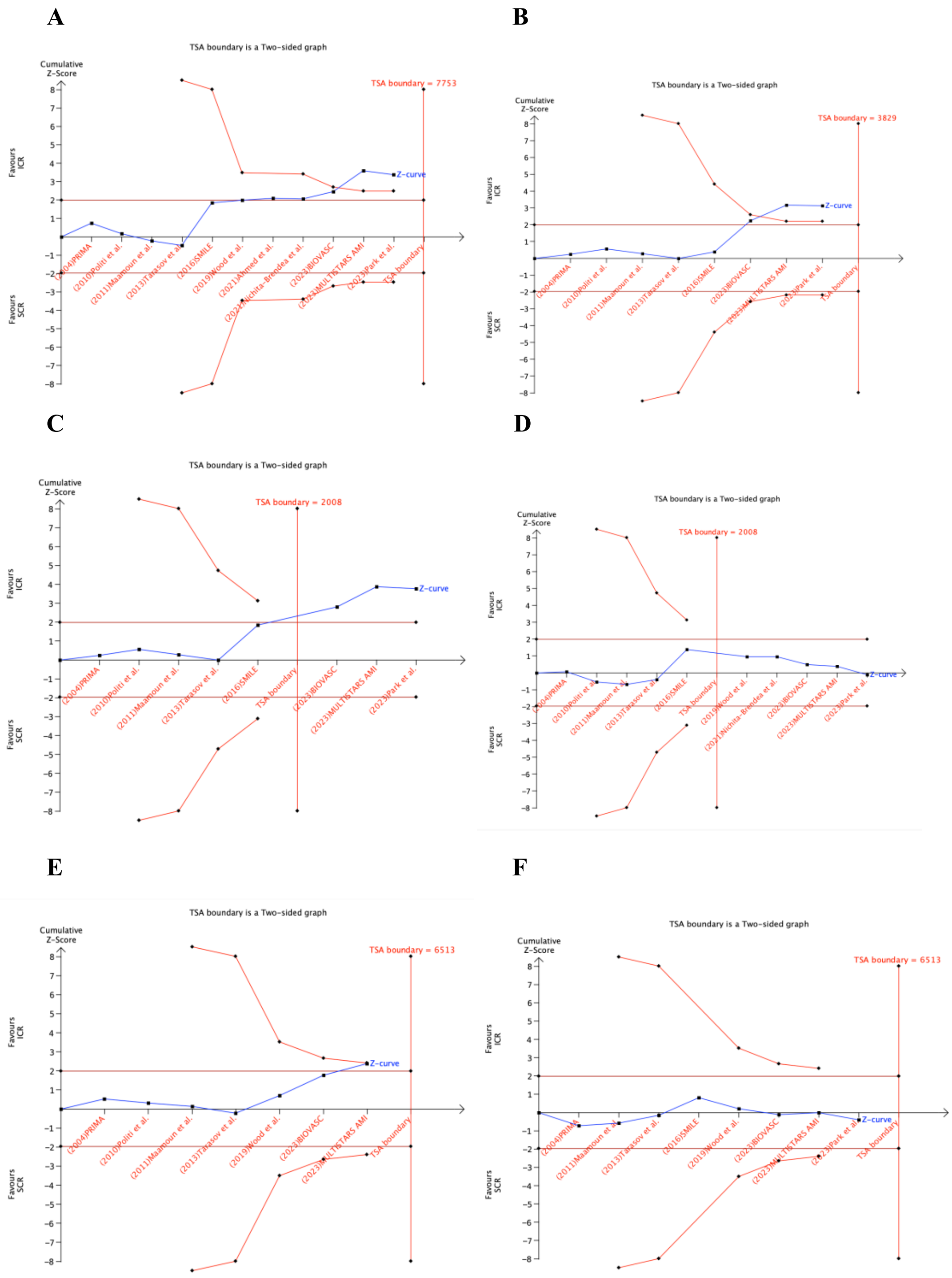

Figure S2. trial sequential analysis.

B. MACE, B.Repeat myocardial infarction; C.repeat revascularization D.all-cause mortality, E.death or myocardial infarction, F.cardiovascular mortality.

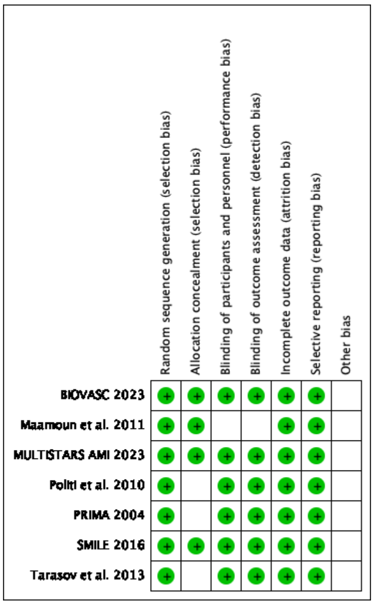

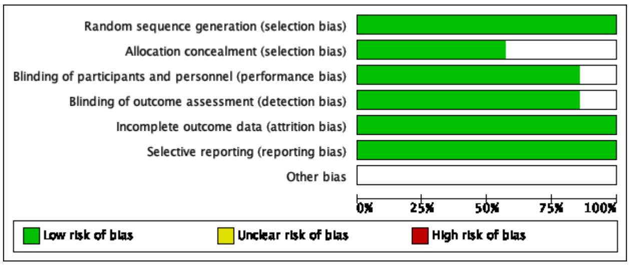

Figure S4. The results of Risk of bias.

Table S3. The result of and certainty of evidence assessment

Certainty assessment						No of patients		Effect	certainty
**No of studies**	**Study design**	**Risk of bias**	**Inconsistency**	**Imprecision**	**Other**	**ICR**	**SCR**	**Relative**	
**Repeat myocardial infraction**									
**8**	Randomized trials	Not serious	moderate	moderate	None	56/1750	91/1740	0.60(0.44-0.83)	moderate
Repeat revascularization									
**8**	Randomized trials	Not serious	moderate	moderate	None	98/1750	154/1740	0.63(0.49-0.80)	moderate
**MACE**									
**11**	Randomized trials	Not serious	moderate	Not serious	None	360/3183	346/2483	0.79(0.68-0.90)	High
**All-cause mortality**									
**10**	Randomized trials	Not serious	Not serious	moderate	None	128/3153	93/2453	1.02(0.78-1.32)	High
Death of myocardial infraction									
7	Randomized trials	Not serious	Not serious	Not serious	None	159/2736	143/2034	0.76(0.61-0.95)	High
Cardiovascular death									
**8**	Randomized trials	Not serious	Not serious	moderate	None	77/3055	52/2359	1.08(0.76-1.53)	High
